# Can visual interpretation of NucliSens graphs reduce the need for repeat viral load testing?

**DOI:** 10.1371/journal.pone.0223597

**Published:** 2019-11-20

**Authors:** Newten Handireketi, Collins Timire, Hemant Deepak Shewade, Ellen Munemo, Charles Nyagupe, Sandra Chipuka, Lucia Sisya, Hlanai Gumbo, Ezekiel Dhitima, Anthony D. Harries

**Affiliations:** 1 Ministry of Health and Child Care Zimbabwe, National Institute of Health Research (NIHR), Harare, Zimbabwe; 2 Ministry of Health and Child Care Zimbabwe, National HIV/AIDS and TB Programme, Harare, Zimbabwe; 3 International Union Against Tuberculosis and Lung Disease, Harare, Zimbabwe; 4 International Union Against Tuberculosis and Lung Disease, New Dehli, India; 5 International Union Against Tuberculosis and Lung Disease, Paris, France; 6 London School of Hygiene and Tropical Medicine, London, United Kingdom; CIRCB - Chantal BIYA International Reference Centre for research on HIV/AIDS prevention and management, CAMEROON

## Abstract

**Background:**

In Zimbabwe, viral load (VL) testing for people living with HIV on antiretroviral therapy is performed at the National Microbiology Reference Laboratory using a NucliSens machine. Anecdotal evidence has shown that invalid graphs for “Target Not Detectable (TND)” will upon repeat VL testing produce a valid result for virus not detected, therefore removing the need to repeat the test. This needs formal assessment.

**Objectives:**

To determine i) intra- and inter-rater agreement of the visual interpretation of NucliSens graphs (Target Detectable [TD], TND and No Line [NL]) between two laboratory scientists and ii) sensitivity, specificity and predictive values of the NucliSens graphs compared with repeat VL results.

**Method:**

Cross sectional study using secondary data. Two laboratory scientists independently rated graphs one week apart for intra-rater agreement and compared final ratings with each other for inter-rater agreement. Consensus interpretations of graphs were compared with repeat VL results. Kappa coefficients were used to obtain measures of agreement.

**Results:**

There were 562 patients with NucliSens graphs and repeat VL. Kappa scores were: 0.98 (Scientist A); 0.99 (Scientist B); 0.96 (Scientist A versus Scientist B); and 0.65 (NucliSens graphs versus VL). Sensitivity, specificity, positive predictive value and negative predictive value for graphs compared with VL were 71%, 92%, 79% and 89% respectively.

**Conclusion:**

Intra-and inter-rater agreements were almost perfect. The negative predictive value translates to a false negative rate of 11%. If repeat VL testing is not done, the clinical consequences need to be balanced against cost savings and the risks outweigh the benefits.

## Introduction

The scale up of antiretroviral therapy (ART) worldwide has been a major public health success story, with 21.7million people accessing ART by the end of 2016[1]. Global coverage of ART reached 59% at the end of 2017, with the largest gains made in the world’s worst affected regions of East and Southern Africa[1]. Zimbabwe has been hard hit by the HIV/AIDS epidemic, but has been one of the high performing Southern African countries with respect to care and treatment. ART was introduced in the public health sector in 2004 and since then there has been a tremendous increase in HIV-infected patients accessing ART with numbers exceeding 900,000 by the end of 2016[2].

In the first few years of ART scale up, the World Health Organization (WHO) recommended that the response to therapy be monitored by clinical assessment and by CD4 cell count[3]. This proved difficult in practice with many false-positive and false-negative results for predicting ART failure[4]. In 2013, and again in 2016, WHO recommended that viral load (VL) testing becomes the standard way to monitor the response to ART[5,6]. Testing should be carried out at 6 months and at every 12 months thereafter to monitor treatment adherence and whether treatment failure has occurred. This move to a viral load monitoring approach opened up the way for the Joint United Nations Program on HIV/AIDS (UNAIDS) to release the 90-90-90 treatment targets for HIV.^7^ These targets specify that by 2020, 90% of people living with HIV will know their HIV status, 90% of people diagnosed as HIV-positive will receive ART and 90% of those on treatment will be virally suppressed.

Zimbabwe has adopted the recent WHO Guidelines and the UNAIDS 90-90-90 targets[6,7]. Since 2013, there has been a massive scale up of VL testing using the NucliSens platform (Biomerieux, France) at the National Microbiology Reference Laboratory (NMRL). However, the implementation of VL testing is not without challenges related to cost and complexity of the testing itself. One of the key challenges is that the proportion of invalid results at the NMRL is around 2.5%. Although this is within the acceptable range (which is 5% according to the manufacturer), the absolute number of invalid results can be high because on average 10 000 tests are performed on the NucliSens platform per month. All invalid results are repeat tested, and the implications for this are threefold.

First, invalid results and repeat testing often increase the laboratory turnaround time from receipt of specimens to production of results to beyond the expected standard of 14 working days. Second, human and material resources are expended in performing the repeat tests and this has cost implications for the HIV/AIDS programme. Third, in some cases the remaining plasma or Dried Blood Sample (DBS) may be insufficient for a repeat test, and the patient may be asked to attend the health facility and submit another sample. Repeat testing for VL therefore comes at a cost to the programme and to the patient.

An invalid VL result is associated with three output graphs, all of which show a non-sigmoidal internal control line and which are stored in the computer of the NucliSens machine. Anecdotal evidence based on visual interpretation of the NucliSens graphs has shown that invalid graphs with “Target Not Detectable (TND)” will upon repeat VL testing almost always produce a valid result for virus not detected. If this anecdotal evidence was proven in a formal study, the laboratory could accept the visual interpretation of an invalid graph for TND as meaning no virus detected and there would be no need to repeat the VL test, saving time, energy and money. According to a PUBMED search, there have been no published studies on this particular subject.

We therefore conducted this study in Zimbabwe to assess whether the visual interpretation of invalid graphs for TND was consistently associated with no virus detected on repeat VL testing. To do this, we obtained records of patients tested for VL between 2013 and 2017. We focused our study on those that initially had invalid results which showed, “Target Detectable (TD)”, TND or no line at all (NL) on the NucliSens graph on the initial test and were subjected to repeat VL testing. Specific objectives were to i) document demographic and laboratory characteristics of HIV patients whose records were included in the study, (ii) assess intra- and inter-rater agreement of the visual interpretation of graphs for TD, TND and NL, and to iii) calculate sensitivity, specificity and predictive values of visual interpretation of graphs compared with repeat VL test results.

## Materials and methods

### Study design

This was a cross-sectional study using routinely collected secondary data.

### Setting

#### General

Zimbabwe is a land-locked country in southern Africa with a population of approximately 13 million and a gross domestic product (GDP) of $924 per capita, which compared with $1,589 per capita for the sub-Saharan Africa region[8,9]. The country has a high burden of HIV/AIDS with an estimated prevalence of 14.6% amongst 15–64 year old adults[2]. Since 2004, when ART was introduced in the public health sector in Zimbabwe, the uptake has increased in a phased and decentralized approach with close to one million people accessing ART country-wide from over 1500 health facilities (>90% of all health facilities in the country)[2].

#### Viral load testing at national microbiology reference laboratory (NMRL)

This study was carried out at the NMRL, Harare, Zimbabwe. The NMRL has conducted all VL testing for the country up to February 2017, at which point VL testing was decentralized to different provinces. All VL testing in NMRL was done using the NucliSens platform (Biomerieux, France) up to February 2017 when Abbott M2000sp and M2000rt machines (Abbott Laboratories, Illinois, USA) were also procured for the laboratory. The majority of samples sent from ART sites from around the country are dried blood spots (DBS) with five spots on each card. The only exception is Harare Central Hospital which sends plasma for VL rather than DBS. This is because the NMRL is located within Harare Central Hospital Complex. The procedures for collecting DBS samples and VL testing are described in the NMRL handbook and standard operating procedures manual[10,11]. The NMRL participated in External Quality Assessment with the Centres for Disease Control and Prevention Global AIDS Program(CDC GAP), receiving 100% accuracy for each of the cycles in which it participated from 2013 to 2017.

Once the VL test has been completed, the machine provides a graphic representation and a printed results summary. Both of these are stored within the machine’s computer system. The printed results summary gives one of the following results: i) invalid result, ii) target virus not detected, or iii) target virus detected with a numeric count of the viral load in copies per ml. As described earlier, all invalid VL results are repeat tested using the remaining samples on the DBS or plasma card.

#### Interpretation of the VL output graphs from the NucliSens machine

The NucliSens machine produces one of five output graphs: two are valid graphs and three are invalid graphs as shown in Figures A-E in S1 Fig.

For a valid graph, the screen always shows a sigmoid line for the internal control. For the tested specimen, any visible graph line means virus is detected (TD) (Fig A in S1 Fig) and no graph line means no virus is detected (TND) (Fig B in S1 Fig).

For an invalid graph, the screen shows no line(NL) at all for the internal control (which indicates no amplification) (Fig C in S1 Fig), non-sigmoidal lines for both the internal control and tested specimen (which indicates incomplete amplification) (Fig D in S1 Fig) or a non-sigmoidal line for the internal control (which indicates incomplete amplification for internal control only) (Fig E in S1 Fig): in these circumstances the summary print out gives “Invalid Result”. For the patient’s tested specimen, any visible graph line means virus is detected (TD) and no graph line means no virus is detected (TND).

#### Study population

HIV-infected people on ART who had an initial invalid NucliSens graph and a repeat VL test in Zimbabwe between 2013 and 2017 were included in the study.

#### Study procedure, intra- and inter-rater variability and comparisons with repeat VL

A line list was made of all the initial invalid VL results that had a repeat VL test. From these, the principal investigator made a list of patients who had graphs (TD, TND and NL). For every TND graph, controls were selected that either had a TD or an NL graph. The graphs were then mixed up. Two similarly experienced medical laboratory scientists (A and B) were trained to interpret the graphs for TND, TD or NL.

Intra-rater agreement: each scientist independently decided on whether the graphs were TD, TND or NL at time T1. The same scientist looked again at the graphs one week later, blinded to what they had decided previously, and decided whether the graphs were TD, TND or NL (T2). The results from T1 and T2 were compared for intra-rater agreement.

Inter-rater agreement: the final decisions on graphs were obtained for each scientist and these were compared between the two to enable inter-rater agreement to be assessed. Each scientist’s final decision was based on whether they agreed on each graph between T1 and T2 –if they had not agreed, they were asked to look for a third time at the graph and make a decision which was accepted as the final decision.

Comparison of graphs with the result of repeat VL testing: When there had been disagreement between the two scientists, a consensus was reached between them on whether the graph was either TD or TND. The concordant results of the two scientists were then compared with the repeat VL printed results–virus detected or virus not detected. The data appears in S1 Dataset.

#### Data variables

Data variables for the study were collected into a structured proforma and included: sex; age; reason for viral load testing; graphs selected by the principal investigator as showing TD, TND and NL; repeat VL printed results that showed virus detected and virus not detected. The data codebook appears in “S2 Dataset. Code book”. The data source was the computer system of the NucliSens platform and VL laboratory electronic information system.

#### Analysis and statistics

Data were double entered from the paper-based form into EpiData (version 4.0.1.44 for data entry and version 2.2.2.186 for data analysis (EpiData Association, Odense, Denmark). These data were exported to STATA (version 13, StataCorp LP, Texas, US).The kappa coefficient (with 95% confidence intervals [CI]) of agreement and its interpretation was used to determine intra- and inter-rater agreement on the visual interpretation of NucliSens graphs (TD, TND and NL) by the two scientists (A and B)[12]. A two-by-two table was constructed to compare the visual interpretation of the graphs (TD and TND) with the repeat VL test result, and this was used to calculate sensitivity, specificity and predictive values.

#### Ethics

Ethics approval was obtained from the Medical Research Council of Zimbabwe and the Ethics Advisory Group, International Union Against Tuberculosis and Lung Disease (The Union) Paris, France. As secondary data were used, a waiver for informed patient consent was obtained from the ethics committees.

## Results

There were 562 patients whose characteristics are shown in Table A in S1 Tables. The majority of patients were aged 18 years or above and in those where gender was recorded the majority was female. All the patients were on ART and had submitted specimens for VL testing as part of follow-up and monitoring. Just over half of the patients had a graph that showed TND, with the remainder divided between TD (17%) and NL (32%). Repeat VL results for the study were only retrieved from the 384 patients who had a graph showing TD and TND.

Intra-rater agreement between scientist A and scientist B in the visual interpretation of invalid NucliSens graphs for TD, TND and NL are shown in Table B in S1 Tables. There was almost perfect agreement, with scientist A having a kappa score of 0.98 between the two readings one week apart and scientist B having a kappa score of 0.99.

Inter-rater agreement between Scientist A and B in the visual interpretation of invalid NucliSens graphs for TD, TND and NL are shown in Table C in S1 Tables. There was an almost perfect agreement with a kappa score of 0.96.

Agreement in the visual interpretation of the NucliSens graphs showing TD and TND and the repeat viral load results showing virus detected and virus not detected are shown in Table D in S1 Tables. There was a substantial agreement with a kappa score of 0.65. Sensitivity, specificity, positive predictive value and negative predictive value for visual interpretation of graphs compared with repeat viral load test results were 71%, 92%, 79% and 89% respectively.

## Discussion

This study confirms that based on the kappa score there was substantial agreement between visual interpretation of the NucliSens graphs showing TND and repeat viral load test results showing virus not detectable. Visual interpretation of the graphs was also straight forward and replicable as indicated by the almost perfect intra-rater and inter-rater agreements when two trained medical laboratory scientists conducted this exercise independently one week apart.

From the point of view of laboratory policy, the key question that needed to be asked was whether the visual interpretation of invalid graphs for TND was consistently associated with no virus detectable and could the laboratory therefore dispense with repeat viral load testing. The high specificity in our study means that 92% of patients with no virus detectable had an invalid graph showing TND. The high negative predictive value means that 89% of invalid graphs showing TND had no virus detectable on repeat VL testing. However, this translates to a false negative rate of 11%; namely that when a medical laboratory scientist concludes that the NucliSens graph of an invalid test shows TND there is in fact detectable virus in the specimen.

Does a false negative rate of 11% matter in terms of laboratory, clinical and programmatic management? In clinical practice, it would mean that 11% of patients on ART would be assessed as having viral suppression when this is not the case and virus is indeed present. The most recent WHO guidelines specify that if virus is present in a patient sample, then a precise VL must be obtained[6]. If the VL is >1000 copies per mL, then adherence counselling has to be done and the patient reassessed three months later with a repeat VL test. If the repeat VL is still high, the patient is judged to have failed the ART regimen which he/she is taking, there might be further tests conducted for HIV drug resistance and the patient would be started on another more suitable ART regimen[6]. If the repeat VL test was not done, none of this clinical and laboratory management would take place and the patient would have to wait for another 12 months until the next repeat VL test is scheduled.

Is it safe to leave a patient on ART with detectable virus for another 12 months before the next repeat VL monitoring test? Probably not! It is well established that detectable virus leads to immunosuppression which increases the risks of further morbidity and mortality. ART with viral suppression effectively prevents the transmission of HIV to other non-infected persons[13,14], and conversely the presence of detectable virus increases the risk of HIV transmission[15]. Thus, detectable virus means that there is a risk that the patient deteriorates clinically as well as transmits HIV to his/ her non-infected partner. HIV drug resistance also needs to be considered, especially for patients already on ART, and unfortunately, HIV drug resistance is a growing threat globally to epidemic control[16]. This is particularly the case in sub-Saharan Africa, where pretreatment resistance to non-nucleoside reverse transcriptase inhibitors in 2016 was greater than 10% in southern and eastern African countries[17]. Detectable virus after the patient has been on ART for some time means there is a risk for drug resistance, and if this is not diagnosed and managed there is further risk of resistance amplification. Thus, the option of leaving clinical staff and patients to believe that virus is suppressed based on visual interpretation of the NucliSens graph, when this is not the case, is not good clinical or programmatic management.

What are the cost savings of this potential approach of accepting invalid TND NucliSens graphs as indicating no virus detected and therefore not repeating the VL test? Working on 10,000 VL tests per month (the approximate workload of the NMRL) with 2.5% being invalid, there would be 250 invalid tests of which half (125) would be TND. Each VL test costs about USD$80 and with none of these TND tests being repeated, there would be a cost savings of USD$10,000 per month or USD$120,000 per annum. For a resource-poor country such as Zimbabwe, this is a considerable cost saving for the NMRL.

The main strengths of the study were the large sample size, the NMRL being the major reference laboratory for the whole country and the rigorous methodology for ascertaining intra- and inter-rater agreements and sensitivity, specificity and predictive values. The NMRL also participates in an external quality assurance programme with excellent results, and therefore the results of VL testing are probably accurate and consistently reproducible. The main limitations were a failure to collect more detailed baseline clinical information on the patients which may have been useful for further analysis and we did not collect precise measurements of VL in copies/mL which would have helped in deciding who had VL above and below 1000 copies/mL on the 11%.

We are unaware of any previous published studies on this subject and therefore cannot compare our findings with those of others. The way forward is for us now to present this data and information to programmatic, clinical and laboratory stakeholders, including those who are responsible for policy decisions within the National AIDS Programme, and decide on what further studies should be done to clarify the situation (such as measuring VL in copies/mL) and on whether or not to dispense with repeat VL testing in those patients for whom an invalid NucliSens graph shows TND. Although there may be considerable cost savings from this approach, there are clinical and programmatic risks, and we feel that we cannot recommend a policy of not repeating the VL if an invalid initial test shows a TND graph.

In conclusion, we evaluated whether the visual interpretation of invalid graphs for TND were consistently associated with no virus detected on repeat VL testing. While we observed excellent intra- and inter-agreement between the two trained medical laboratory scientists in visual interpretation of the graphs, the specificity and negative predictive values of TD and TND graphs against repeat VL test results were 92% and 89% respectively. The false negative rate of 11% with its programmatic and clinical consequences has to be balanced against the cost savings of dispensing with the repeat VL test. On balance the risks outweigh the benefits and we cannot recommend a policy of no repeat VL testing.

## Supporting information

S1 Fig (DOCX)

S1 Tables (DOCX)

S1 Dataset (ZIP)

S2 Dataset Code book. (ZIP)
