# Supplementary figures and images for: Can visual interpretation of NucliSens graphs reduce the need for repeat viral load testing?
Source: PLoS One. 2019 Nov 20;14(11):e0223597. doi: 10.1371/journal.pone.0223597 (PMC6867593; doi:10.1371/journal.pone.0223597)

**S1 Fig**


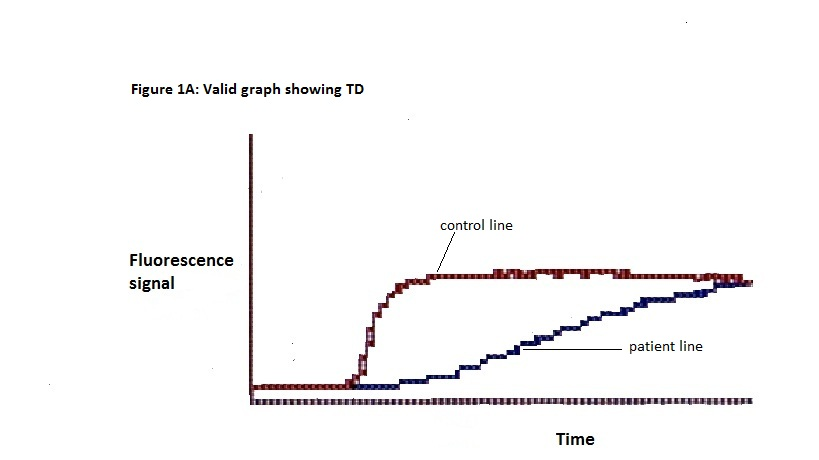


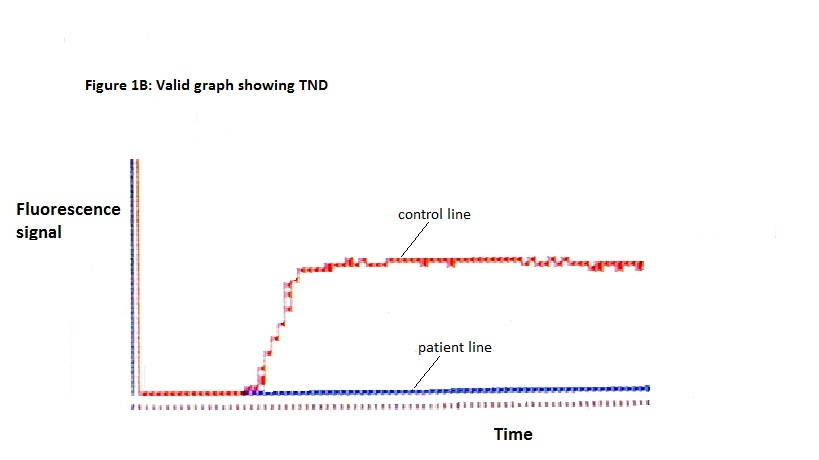


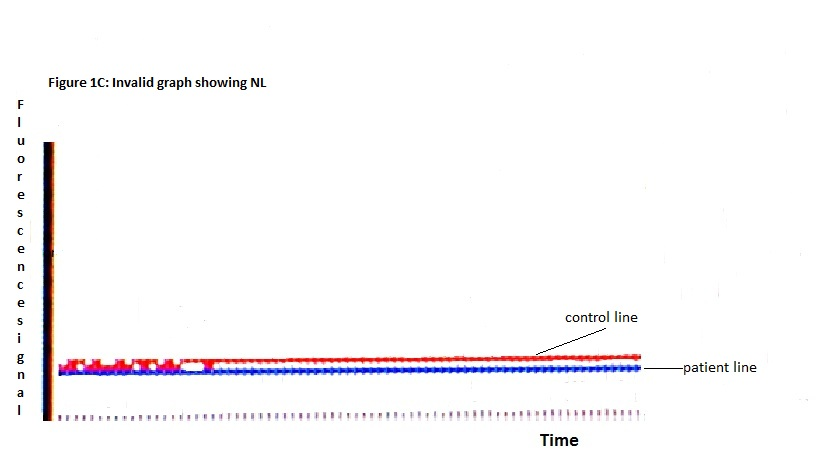


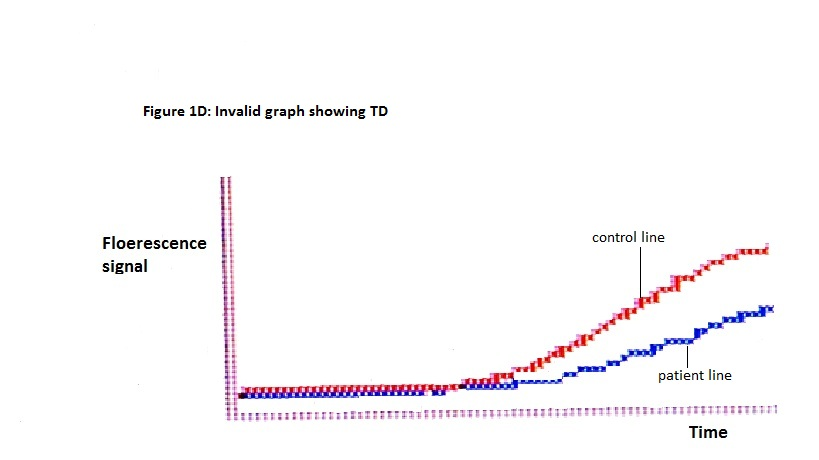


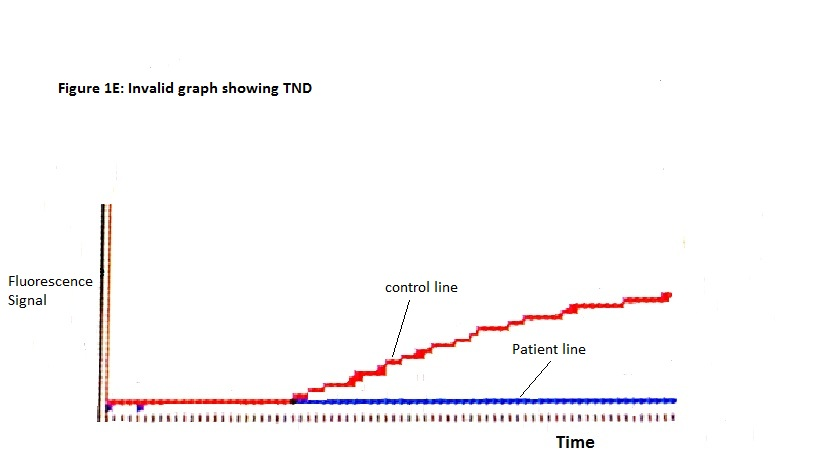

Supplement: S1 Fig — (DOCX) [file pone.0223597.s001.docx]
