# Supplementary material for: Can visual interpretation of NucliSens graphs reduce the need for repeat viral load testing?
Source: PLoS One. 2019 Nov 20;14(11):e0223597. doi: 10.1371/journal.pone.0223597 (PMC6867593; doi:10.1371/journal.pone.0223597)
Supplement: S1 Tables — (DOCX) [file pone.0223597.s002.docx]

**S1 Tables**

**Table 1: Characteristics of HIV-infected people on ART in Zimbabwe who had an initial invalid NucliSens graph and a repeat VL test: 2013 – 2017**

| Characteristic | n | (%) |
| --- | --- | --- |
| All HIV-infected people | 562 |  |
| Age group (years): |  |  |
| <18 | 17 | (3) |
| ≥18 | 534 | (95) |
| Not recorded | 11) | (2) |
| Gender: |  |  |
| Male | 151 | (27) |
| Female | 312 | (55) |
| Not recorded | 99 | (18) |
| Visual Interpretation of Graphs: ^a^ |  |  |
| TD | 99 | (17) |
| TND | 285 | (51) |
| NL | 178 | (32) |
| Repeat VL test result: ^b^ |  |  |
| Virus detected | 110 | (29) |
| Virus not detected | 274 | (71) |

TD = target detectable; TND = target not detectable; NL = no line at all on NucliSens graph

^a^The visual interpretation of the NucliSens graphs shown here was based on the final consensus rating of the two laboratory scientists A and B.

^b^For the purpose of the study, results of repeat viral load testing were only retrieved for the graphs which were rated as TD or TND from visual interpretation. There was no point in comparisons for NL graphs as these showed no lines at all and therefore there was no possibility for visual misinterpretation.

**Table 2: Intra-rater agreement in the visual interpretation of invalid NucliSens graphs for TD, TND and NL by scientists A and B at times T1 and T2 at the National Microbiology Reference Laboratory, Harare, Zimbabwe: 2013-2017**

| A | | | | | | |
| --- | --- | --- | --- | --- | --- | --- |
|  | Visual Interpretation of Graph at T2 | | | | | Total |
|  |  | TD | TND | | NL |  |
| Visual Interpretation of Graph at T1 | TD | 90 | 0 | | 2 | 92 |
|  | TND | 2 | 283 | | 3 | 288 |
|  | NL | 0 | 1 | | 181 | 182 |
|  | Total | 92 | 284 | | 186 | 562 |
| B | | | | | | |
|  | Visual Interpretation of Graph at T2 | | | | | Total |
|  |  | TD | TND | NL | |  |
| Visual Interpretation of Graph at T1 | TD | 92 | 0 | 0 | | 92 |
|  | TND | 0 | 284 | 1 | | 285 |
|  | NL | 1 | 2 | 182 | | 185 |
|  | Total | 93 | 286 | 183 | | 562 |

Intra-rater agreement for Scientist A at T1 and T2 Kappa score was 0.98(95% CI, 0.92 - 1.00)

Intra-rater agreement for Scientist B at T1 and T2 Kappa score was 0.99(95% CI, 0.93-1.00)

TND- Target Not Detectable; TD- Target Detectable; NL = no line at all on NucliSens graph;

T1 = first visual interpretation by Scientist A and B

T2 = second visual interpretation by Scientist A and B one week later

**Table 3: Inter-rater agreement between Scientist A and B in the visual interpretation of invalid NucliSens graphs for TD, TND and NL at the National Microbiology Reference Laboratory, Harare, Zimbabwe: 2013-2017**

|  | Visual Interpretation of Graph by Scientist B | | | | Total |
| --- | --- | --- | --- | --- | --- |
|  |  | TD | TND | NL |  |
| Visual Interpretation of Graph by Scientist A | TD | 87 | 2 | 4 | 93 |
|  | TND | 4 | 283 | 1 | 288 |
|  | NL | 3 | 1 | 177 | 181 |
|  | Total | 94 | 286 | 182 | 562 |

Kappa score was 0.96(95% CI, 0.89 - 1.00)

TND- Target Not Detectable; TD- Target Detectable; NL = no line at all on NucliSens graph;

**Table 4: Agreement in the visual interpretation of the NucliSens graphs and repeat viral load results at the National Microbiology Reference Laboratory, Harare, Zimbabwe: 2013-2017.**

| Visual Interpretation of Graph | Repeat Printed VL result | | | Total |
| --- | --- | --- | --- | --- |
|  |  | Virus detected | Virus Not detected |  |
|  | TD | 78 | 21 | 99 |
|  | TND | 32 | 253 | 285 |
|  | Total | 110 | 274 | 384 |

Kappa score was 0.65(95% CI, 0.55 - 0.75)

VL= viral load; TD= Target detectable; TND=Target not detectable

Calculations of sensitivity, specificity, positive and negative predictive values (PPV / NPV)

Sensitivity (78/110)*100=71% (95% CI, 61% - 79%)

Specificity (253/274)*100=92% (95% CI, 89% - 95%)

PPV (78/99)*100=79% (95% CI, 71% - 85%)

NPV (253/285)*100=89% (95% CI, 85% - 91%)
