# Supplementary material for: Can visual interpretation of NucliSens graphs reduce the need for repeat viral load testing?
Source: PLoS One. 2019 Nov 20;14(11):e0223597. doi: 10.1371/journal.pone.0223597 (PMC6867593; doi:10.1371/journal.pone.0223597)
Supplement: S1 Dataset — (ZIP) [file pone.0223597.s003.zip › S3Dataset Code book_v1.docx]

**Code book/ Data documentation Sheet.**

| Field name | Field label | Field type | Field length | Field values | Value labels | Comments |
| --- | --- | --- | --- | --- | --- | --- |
| labidno | unique laboratory number of each patient | Integer | 6 | 1-300000  300001, 300002. |  | The unique lab number from 1 to 300 000  Reserve and assign (300001,300002..) if missing; Make a note during data entry |
| sex | The sex of the patient | Integer | 1 | 1  2  9 | Female  Male  Not entered |  |
| agecat | The age category of the patient | Integer | 1 | 1  2  9 | >=18 years  <18 years  Not entered |  |
| rslt1ra | Rater A result at time t1 | Integer | 1 | 0  1  9 | TND  TD  Not recorded |  |
| rslt2ra | Rater A result at time t2 | Integer | 1 | 0  1  9 | TND  TD  Not recorded |  |
| rslt1rb | Rater B result at time t1 | Integer | 1 | 0  1  9 | TND  TD  Not recorded |  |
| rslt2rb | Rater B result at time t2 | Integer | 1 | 0  1  9 | TND  TD  Not recorded |  |
| consres | consensus rating | Integer | 1 | 0  1  9 | TND  TD  Not recorded |  |
| rptesres | repeat VL result from machine | Integer | 1 | 1  2  9 | TND  TD  Not recorded |  |
